# Coupling Capillary Electrophoresis With a Shifted Inlet Potential High‐Resolution Ion Mobility Spectrometer

**DOI:** 10.1002/elps.8147

**Published:** 2025-04-28

**Authors:** Klaus Welters, Christian Thoben, Christian‐Robert Raddatz, Florian Schlottmann, Stefan Zimmermann, Detlev Belder

**Affiliations:** ^1^ Institute of Analytical Chemistry Leipzig University Leipzig Germany; ^2^ Department of Sensors and Measurement Technology, Institute of Electrical Engineering and Measurement Technology Leibniz University Hannover Hannover Germany

**Keywords:** collision cross section, hyphenation, ion mobility spectrometry, nanoflow electrospray

## Abstract

We present the coupling of capillary electrophoresis to a custom‐built high‐resolution ion mobility spectrometer (IMS). This system integrates a shifted inlet potential IMS configuration with a customised nanoflow ESI sheath interface. It enables the rapid analysis of quaternary ammonium compounds (QACs) and their impurities in real‐world samples. It allowed the detection of six non‐chromophoric compounds in about 3 min. The assignment of the IMS signals to compounds was supported by matching experimentally determined collision cross‐section (CCS) values with predicted values. The system achieved a detection limit in the single‐digit picogram range with IMS resolutions of over 80.

## 1 Introduction

Ion mobility spectrometry (IMS) leverages the interaction of ions with a neutral gas phase under the influence of an electric field to separate ions based on their specific mobility. The method has evolved significantly since its foundational studies in the 1950s [1], gaining traction through the first hyphenations with gas chromatography in the 1970s and later with other separation techniques like capillary electrophoresis (CE) in the late 1980s [2, 3]. More recently, IMS has garnered widespread attention due to its integration into mass spectrometers (MS), resulting in the widespread commercial adoption of ion mobility‐mass spectrometry (IM‐MS) platforms. This combination has proven valuable in various fields, including proteomics and glycomics, where it serves as an additional dimension of separation prior to mass analysis [4, 5]. The growing popularity of IM‐MS has led to a surge in the development of experimental collision cross‐section (CCS) databases and advanced prediction models, which aim to expand the utility of CCS values as a widely available and informative data dimension [6, 7, 8, 9, 10]. While these advancements have enhanced the capabilities of IM‐MS, these systems are not ideal for all applications due to the need for high‐vacuum conditions, which increase costs, limit portability and affect robustness. These challenges highlight the advantages of standalone IMS detectors, which have established themselves as valuable tools for rapid screening, especially in security‐sensitive areas such as detecting illicit drugs, explosives and chemical warfare agents [11, 12, 13, 14]. Beyond these applications, IMS was also presented for applications such as medical biomarker monitoring, food safety and, more recently, the analysis of biogas [15, 16, 17]. While many IMS applications focus on volatile analytes and gaseous sample introduction methods, liquid‐based approaches have also been explored. Combining electrophoresis in solution with IMS has been a longstanding area of research. Although CE and IMS leverage similar properties, namely the motion of ions through a medium under the influence of an electric field, the differences afforded by their respective media allow for a useful amount of orthogonality [18, 19].

Pioneering efforts by the Hill group demonstrated the fundamental feasibility of coupling CE to IMS by various interfaces [3]. Although much of the subsequent research focused on coupling CE with commercial IM‐MS systems [20, 21, 22], hyphenation with standalone IMS systems has also been explored: Guo et al. demonstrated multiplexing approaches in CE‐IMS using tetraalkylammonium (TAA) standards [23], and Masar et al. coupled microchip electrophoresis to IMS via a ‘direct liquid sampling’ interface and corona discharge ionisation for the analysis of alkanoic acids [24]. Hartner et al. further enhanced separation orthogonality by interfacing chip electrochromatography with ESI‐IMS [25]. Although such chip‐based approaches offer numerous advantages, they are not yet standard procedure within the laboratory setting.

To this end, the present work describes a novel high‐resolution IMS with shifted potentials for straightforward coupling with commercial CE instruments with a tailored nanoflow ESI interface. The miniaturisation and integration of CE and IMS functionalities offer promising prospects for future point‐of‐care devices for complex mixture analysis in solution.

## 2 Materials and Methods

### 2.1 Chemicals

Acetonitrile (ACN, HiPerSolv HPLC grade) was purchased from VWR Int. GmbH (Darmstadt, Germany), while sodium hydroxide (NaOH), sodium acetate (NaAc, water‐free) and acetic acid (HAc, 96%) as well as tetrabutyl‐ (TBA), tetrahexyl‐ (THA) and tetraoctylammonium bromide (TOA) (all >97%) were obtained from Merck KGaA (Darmstadt, Germany). Water was purified using a TKA Smart2Pure purification system (<0.055 µS/cm, TKA GmbH, Niederelbert, Germany). The rinse‐off hair conditioners sample A, ‘Balea Seidenglanz’ and sample B, ‘Balea Silberglanz’ (both from dm‐drogerie markt GmbH, Karlsruhe, Germany), were bought locally in a drug store. Samples of hexadecyltrimethylammonium chloride (Cetrimonium, Cet) (95%, abcr GmbH, Karlsruhe, Germany) and dodecyltrimethylammonium chloride (Behentrimonium, Beh) (80%, BLD Pharmatech GmbH, Kaiserslautern, Germany) were purchased, while N‐(2‐Hydroxyethyl)‐N‐methyl‐2‐[(1‐oxooctadecyl)oxy]‐N‐[2‐[(1‐oxooctadecyl)oxy]ethyl]‐ethanaminium methyl sulphate (distearoylethyl hydroxyethylmonium methosulphate, DHM) was received as a sample free of charge (Dehyquart F 75 T, BASF SE, Ludwigshafen, Germany).

### 2.2 Instrumentation

#### 2.2.1 Capillary Electrophoresis System

CE separations were performed using a G1600AX CE system (Agilent GmbH, Waldbronn, Germany) equipped with a CE‐MS compatible capillary cassette. This configuration allowed for direct connection of the capillary to the inlet vial while routing the capillary and its outlet outside the instrument enclosure. New CE capillaries were initially conditioned by automated flushing at 1 bar pressure using the following sequence: 5 min of 0.1 M NaOH, 3 min of pure water and 5 min of background electrolyte (BGE). All samples were injected hydrodynamically at 50 mbar for 10 s, unless stated otherwise. Separations were performed at a constant temperature of 25°C. A post‐run conditioning step involving a 5‐min BGE flush was implemented after each analysis. Fused silica (FS) capillaries (50 µm ID, 363 µm OD, Molex Polymicro TSP, Lisle, IL, USA) were cut to a length of 68 cm. The outlet end of the FS capillary was manually tapered by grinding at a 9.5° angle, resulting in an OD at the tip of approximately 60 µm.

#### 2.2.2 Sheath Liquid Setup

**FIGURE 1:**
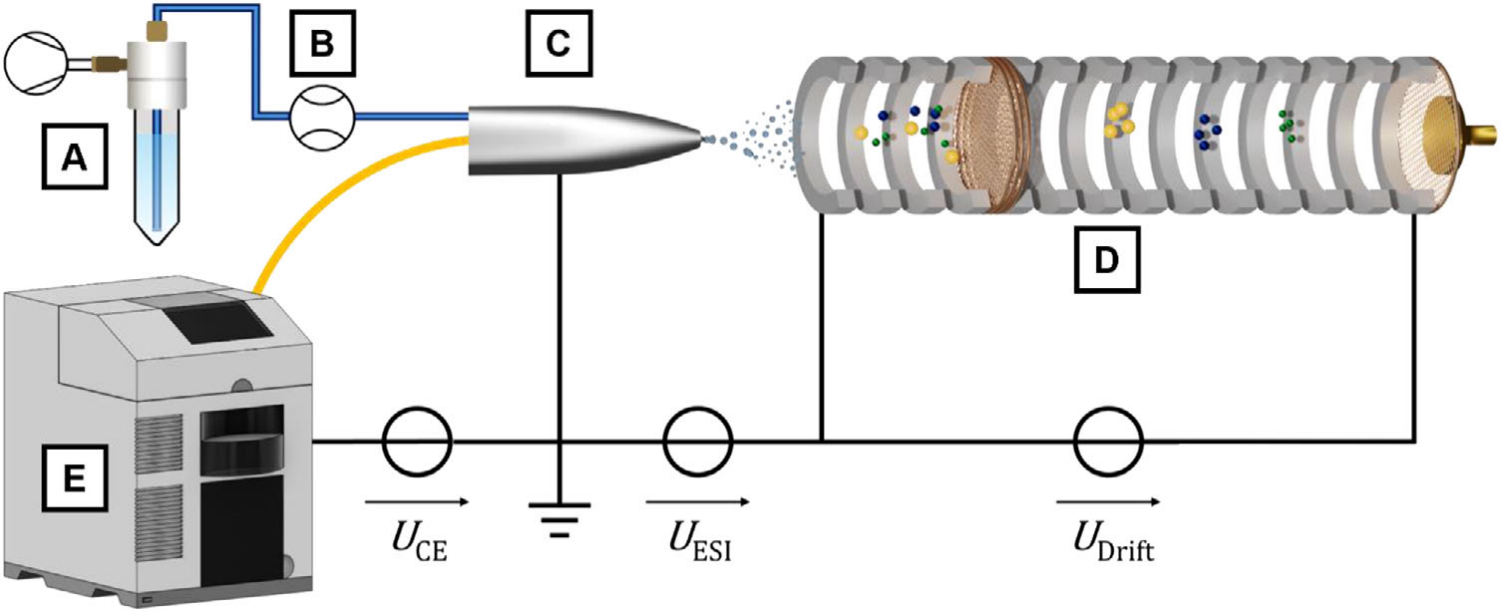
Schematic of the instrumental setup with voltage gradient. (A) Pressure driven sheath liquid pump with reservoir. (B) Sheath liquid flow sensor. (C) Grounded nanoflow sheath liquid electrospray interface. (D) Drift tube ion mobility spectrometer (DT‐IMS). (E) Commercial capillary electrophoresis instrument.

**FIGURE 2:**
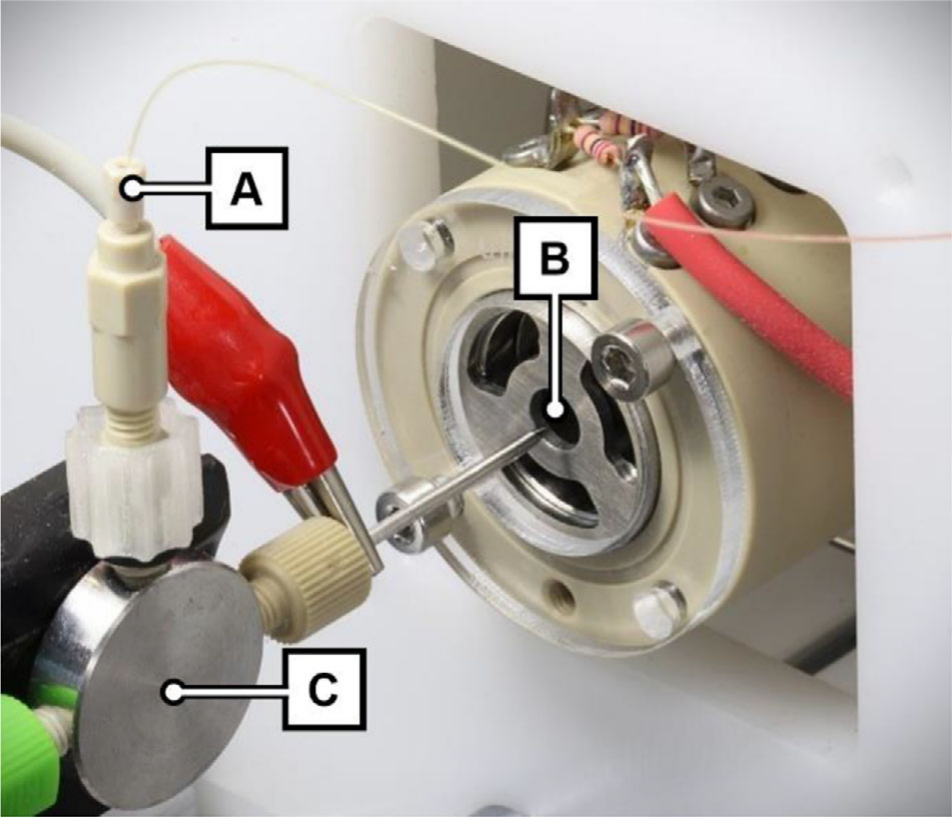
Photograph of the IMS and the nanoflow sheath liquid ESI interface. (A) Sheath liquid delivery line. (B) IMS sample inlet with electrostatic lens. (C) T‐piece passthrough and interface emitter tip with enclosed separation capillary and ground contact clamp.

The sheath liquid consisted of an 8/2 (v/v) mixture of ACN/H_2_O, delivered by a pressure‐based flow controller system (Fluigent SAS, Le Kremlin‐Bicêtre, France). A flow sensor unit with a custom calibration set in the proprietary OxyGEN software was used as a control. The flow rate was set to 600 nL/min for flushing and injection steps and, upon triggering by the CE instrument, lowered to 400 nL/min while the separation voltage was applied (Figure 1). This sheath liquid flow was connected to the orthogonal connection of a stainless steel (SST) T‐piece (ZT1, Vici AG, Schenkon, Switzerland). The tapered outlet end of the FS separation capillary was guided through the inline ports of the T‐piece and an attached tapered SST capillary (380 µm ID, 1/16″ OD, VICI). By extending the FS capillary approximately 0.1 mm beyond the SST capillary, the emitter section of the nanoflow sheath liquid ESI interface is formed and subsequently positioned in front of the IMS inlet. A contact clamp, connected to the SST capillary and referenced to ground potential, was used to close both CE and ESI circuits electrically. The nanoflow sheath liquid ESI interface was positioned relative to the IMS inlet employing a manual stage (T12XYZ, Thorlabs GmbH, Bergkirchen, Germany) and is shown in Figure 2.

#### 2.2.3 Ion Mobility Spectrometry

In this work, Zimmermann's group well‐established high‐resolution drift tube IMS [26] was modified by incorporating two custom‐built, isolated 10 kV power supplies. The resulting system retained the core functionalities of its predecessor, including ambient pressure and temperature operation (Table 1) and the incorporation of a tristate ion shutter [27], while introducing a novel capability: the ability to ground the ionisation source, such as an electrospray emitter. This was achieved by independently controlling the inlet and desolvation/drift field potentials. By connecting two custom‐built isolated high‐voltage power supplies in series, the first power supply established the high ground reference for the second. The first power supply controlled the inlet potential of the IMS, while the second regulated the potential difference over the desolvation and drift regions. As a result, the detector, amplifier and data acquisition components operated at a high potential [28]. Therefore, a self‐designed and self‐constructed isolated DC power supply with 50 kV isolation and a high overall efficiency of 82.5% at 55 W is used to supply the electronics [29]. This configuration enabled flexible electrospray grounding in both positive and negative ion modes (Figure 1). The drift times (*t*_d_) yielded by measurements were converted to inverse reduced mobility values (1/*K*_0_) according to Equation 1, using data obtained from the device's integrated temperature (*T*) and pressure (*p*) sensors as well as the drift region length (*L*) and potential (Δ*U*):

(1) $$\frac{1}{K_{0}}={\left(\frac{L^{2}}{\Delta U\cdot t_{d}}\cdot \frac{p}{p_{0}}\cdot \frac{T_{0}}{T}\right)}^{-1}$$

**FIGURE 3:**
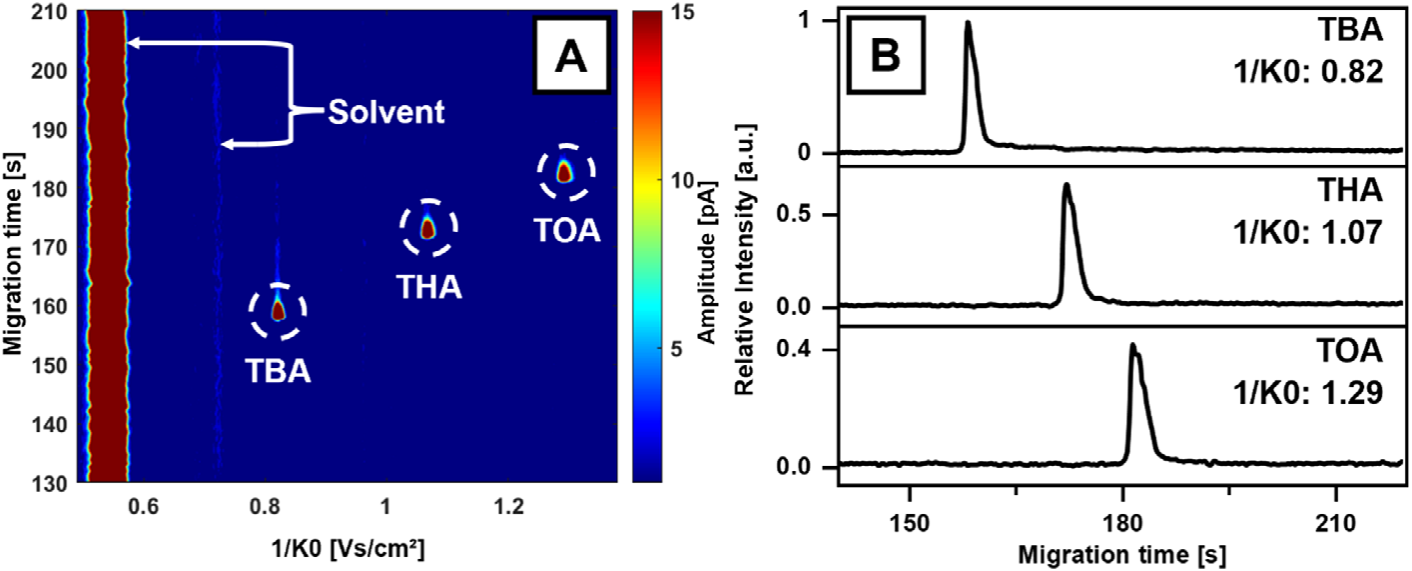
CE‐IMS separation of tetrabutyl‐ (TBA), tetrahexyl‐ (THA) and tetraoctylammonium bromide (TOA) in positive ion mode. (A) 2D plot of signal intensity plotted against migration time and inverse reduced ion mobility. (B) Electropherograms of the three analytes, obtained by integration over a ±13.3 mV·s/cm^2^ interval for the characteristic mobilities of the analytes as indicated by maximum peak intensity. Intensity normalized to peak intensity of TBA. Reduced ion mobility values (*K*_0_) of signals in cm^2^/(V·s): TBA: 1.219; THA: 0.938; TOA: 0.775. CE parameters: separation voltage: 30.0 kV, capillary length: 68 cm, sheath flow: 400 nL/min 8/2 ACN/H_2_O (v/v), BGE: 8/2 ACN/H_2_O (v/v) + 1 mM NaAc/HAc pH 4, ESI voltage +3.5 kV, sheath flow 400 nL/min 8/2 ACN/H_2_O (v/v).

**FIGURE 4:**
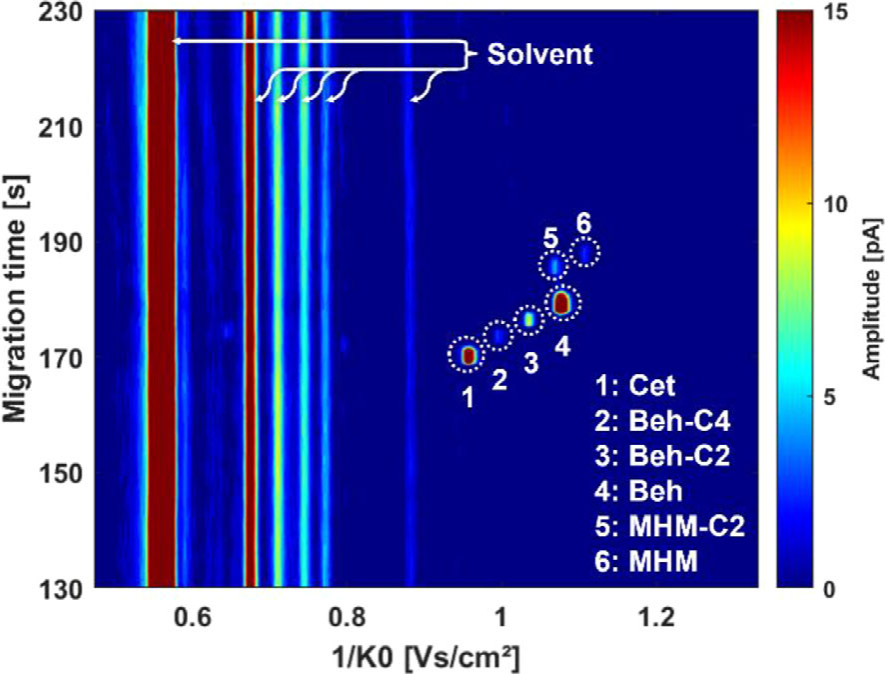
2D plot of a CE‐IMS separation run of sample B at a dilution of 5E‐4 in positive ion mode with signals assigned to the analytes of interest, signal intensity plotted against migration time and inverse reduced ion mobility. Reduced ion mobility values (*K*_0_) of signals in cm^2^/(V·s): 1 (Cet) 1.043; 2 (Beh‐C4) 1.002; 3 (Beh‐C2) 0.965; 4 (Beh) 0.928; 5 (MHM‐C2) 0.935; 6 (MHM) 0.901. CE parameters: separation voltage: 26.0 kV, capillary length: 68 cm, sheath flow: 400 nL/min 8/2 ACN/H_2_O (v/v), BGE: 8/2 ACN/H_2_O (v/v) + 1 mM NaAc/HAc pH 4, ESI voltage +3.5 kV, sheath flow 400 nL/min 8/2 ACN/H_2_O (v/v).

System control was implemented using LabVIEW 2018 (National Instruments, Austin, TX, USA), while data analysis was performed with MATLAB (version R2022a, Mathworks, Natick, MA, USA) and OriginPro 2019 (OriginLab Corporation, Northampton, MA, USA). The acquired ion mobility spectra, structured as reduced ion mobility versus signal amplitude, were first processed using a 10 kHz low‐pass filter. To incorporate migration time as a third dimension, all individual spectra of a selected migration time frame were sequentially plotted, with signal amplitude represented by colour. This resulted in the generation of 2D heatmap plots such as Figures 3A and 4. The data used for electropherograms was processed using a two‐point window FFT filter, while the ion mobility spectra used for display were background‐corrected and filtered using a twenty‐point window moving average. The data used for 2D heatmap plots was filtered in the ion mobility domain with a three‐point median filter.

#### 2.2.4 Mass Spectrometry

A micrOTOF‐Q III mass spectrometer (Bruker Daltonics GmbH, Bremen, Germany) was coupled to the CE system using a CE ESI triple tube sprayer (Agilent P/N G1607‐60001) mounted on the standard Bruker ESI spray chamber and used in standard (non‐nanoflow) mode. CE conditions were maintained identical to those employed in the CE‐IMS experiments, while the sheath flow was set to 5 µL/min at a nebuliser pressure of 15 psi. The mass spectrometer was operated in positive ion mode with a dry gas flow of 3.0 L/min at 200°C and a spray voltage of +4.0 kV. Data analysis was conducted using Data Analysis (Bruker Daltonics) and OriginPro software.

**TABLE 1:** IMS operating parameters.

*U*_ESI_ Source/Inlet	0/−3.5 kV	*f*_Acq. max_	10 Hz
*U*_Drift/Desolv_	−9.6 kV	*L*_Drift_	77 mm
*t*_inj_	20 µs	*L*_Desolvation_	76 mm
*t*_d_ monitored	5.5–15.5 ms	*Q*_Nitrogen_	250 mL/min
*T*_Tube_	297–304 K	*p*_ambient_	1005–1030 hPa

### 2.3 Background Electrolyte and Sample Preparation

A 100 mM NaAc/HAc buffer was prepared by dissolving 410.4 mg NaAc in 45 mL water in a 50 mL volumetric flask, adding 317 µL HAc and diluting to volume with water. This solution was further diluted to 5 mM and adjusted to pH 4.0 using HAc while stirring and monitoring pH with a Lab 850 pH meter (SI Analytics GmbH, Mainz, Germany). The final BGE was obtained by mixing this buffer with four volumes of ACN, resulting in an 8:2 (v/v) ACN/H_2_O composition and 1 mM concentration of NaAc/HAc buffer.

Individual 30 µM solutions of TBA, THA and TOA were prepared in an 8:2 (v/v) ACN/H_2_O solvent. These solutions were mixed in equal proportions to create a mixed standard of 10 µm concentration. Stock solutions of hair conditioners sample A (‘Seidenglanz’) and B (‘Silberglanz’) were prepared by dissolving 181 and 197 mg, respectively, in 17.92 and 19.50 mL water, yielding approximate concentrations of 1/100 (w/v). For analysis, 50 µL of each stock solution was diluted with 150 µL water and 800 µL ACN to achieve a final concentration of 1/2000 (w/v) in an 8:2 (v/v) ACN/water mixture. After sonication for 3 min, these diluted samples were transferred to 2 mL glass vials and directly loaded into the CE instrument without further preparation.

## 3 Results and Discussion

The recent development of shifted potential ion mobility spectrometers (IMS) [30] has facilitated the direct coupling of these devices with miniaturised systems with integrated electrospray emitters, such as chip electrochromatography and chip HPLC [25, 31]. Previously, the inlet potential of these ESI‐IMS was at ground potential, so the ESI emitter had to be at an elevated potential of several kV. Further development of this concept led to the system presented herein: By employing a dedicated variable and reversible high‐voltage source for the inlet potential while maintaining a constant drift region potential, the IMS can be used in either ion mode regardless of the polarity or potential of the ion source. This is particularly advantageous for coupling CE with IMS since, in related CE‐MS systems, those couplings have become established in which the ESI emitter is also at ground potential. Accordingly, this work focuses on coupling a CE system with an advanced IMS featuring a shifted inlet potential configuration and evaluating its potential for practical analytical applications. From different perspectives, the IMS can be seen as a compact, cost‐effective detector for CE, while CE is an ideal pre‐separation module for the IMS. The coupling of CE with IMS presents a compelling multidimensional analysis technique that does not require complex pump technology, as it works at ambient conditions.

### 3.1 Electrospray Interface

Utilising an IMS with isolated data acquisition and adjustable drift potential allowed for grounding the ESI ion source, safely decoupling separation and ionisation potentials. This configuration facilitates significantly higher effective electrophoretic separation voltages and thus achievable separation speeds compared to the state‐of‐the‐art CE‐IMS coupling [3, 23]. In addition, incorporating an ionisation interface with a grounded emitter enhances user and instrument safety, prevents electrolysis and economises an external high‐voltage source.

The design of the ESI interface, transferring analytes from the liquid to ions in the gas phase, is essential for CE‐MS and IMS couplings. It must ensure a reliable electrical contact to build up the electric field between the capillary inlet and outlet for electrophoresis and simultaneously function as an electrospray emitter. Over time, numerous interfaces have been proposed, demonstrated and commercialised, utilising different approaches. While some apply direct electrical contact to the CE capillary outlet [32], the established sheath‐flow approach is employed for conventional FS CE capillaries that are not electrically conductive. In this setup, the CE capillary is inserted into a larger capillary, and electrically connected sheath liquid is pumped into the space between them to close the electrical circuit at the capillary end [33, 34, 35, 36]. For these sheath liquid‐based methods, reducing the flow of the sheath liquid has been a key objective, as this minimises sample dilution during the electrospray process, thereby improving sensitivity. Such approaches typically apply flow rates in the sub‐microliter per minute range.

To achieve minimal sheath liquid‐induced dilution, we constructed a tailored interface: The design incorporates an SST sheath capillary with an ID of 380 µm, which is only marginally larger than the OD of 363 µm of the CE capillary positioned inside. The resulting gap filled with sheath liquid is thereby minimised, thus reducing contact resistance and enhancing spray robustness at very low flow rates. The tip of the separation capillary was tapered, further enhancing the electrospray process at low flow rates. This was inspired by triple‐tube sprayer approaches described earlier [37]. Our further miniaturised setup achieved comparable sensitivities and improved spray stability at sheath liquid flow rates below 600 nL/min with relative standard deviations (RSDs) of about 5% (see Figure S2). The nanoflow electrospray was continuously monitored using a digital microscope while illuminating the spray with green laser light, as shown in Figure S1. It showed a high degree of stability, greatly benefiting IMS signal quality.

### 3.2 Sample Analysis

Quaternary ammonium compounds (QAC) are commonly used as biocides, preservatives, cleaning agents and in cosmetics [38]. Due to environmental and health concerns with traditional QACs [39, 40, 41], ester quats were developed, featuring ester‐linked alkyl chains to improve biodegradability [42, 43]. Analytical techniques leveraging the ionic nature of QACs are particularly effective for their detection. A mixture of three QACs commonly used as IMS standards, TBA, THA and TOA, was analysed at a concentration of 10 µM to test and evaluate the function of the Nanoflow‐CE‐ESI coupling with IMS. As seen in Figure 3, a baseline separation of all three compounds in both drift time and electrophoretic migration time dimensions was successful. An IMS resolving power of *R*_P_ = 77.4–86.4 was achieved based on full width at half maximum (FWHM) calculation criteria.

In addition to the three distinct analyte signals, the spectrum in Figure 3A exhibits a prominent, continuous signal at an inverse reduced ion mobility of approximately 0.55 V·s/cm^2^, accompanied by a signal of low intensity at 0.68 V·s/cm^2^. These signals are attributed to the solvent and BGE, likely arising from positively charged mono‐ and multimers of the organic solvent component. Integration of drift time intensities within a ±13.3 mV·s/cm^2^ interval centred on each peak maximum yielded the analyte ions’ traces, subsequently visualised as electropherograms in Figure 3B. The reduced ion mobility values *K*_0_ obtained in this measurement for the TAA standards are in accordance with those reported in the literature [44].

**FIGURE 5:**
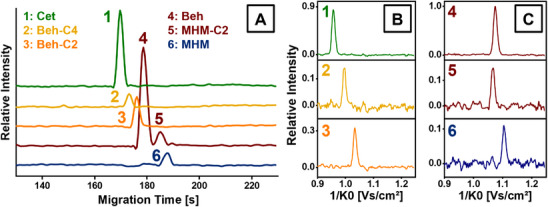
CE‐IMS separation run of sample B at a dilution of 5E‐4 in positive ion mode. (A) Electropherograms of the analytes’ characteristic mobility trace intensities plotted against the migration times, obtained by integration over a ±13.3 mV·s/cm^2^ interval maximum peak intensity. Signals #4 and #5 are displayed in the same trace in the electropherogram due to similar mobilities. (B) and (C) Details of the individual ion mobility spectra (background subtracted) shown at migration times of respective maximum peak intensity for all six QAC signals, relative intensity normalized to the peak intensity of Beh (signal #4).

Building on these results, further analysis of the two cosmetic products, Samples A and B, was conducted employing the same method. The target analytes for the cosmetic products were originally the QAC declared in the INCI ingredient lists of both products: Cet, Beh and DHM (see Table S1). Employing the CE‐hyphenated IMS, we were able to fully separate all signals, achieving IMS resolutions ranging from *R*_P_ = 80.4 to 90.7. While the signals for Cet and Beh were quickly identified at inverse reduced ion mobilities of ca. 0.96 and 1.08 V·s/cm^2^ (Signals 1 and 4 in Figure 4), the occurrence of additional signals with similar mobilities and migration times as well as the absence of the DHM signal prompted further analysis. The signals labelled 2 and 3 in Figure 3 at inverse reduced ion mobilities of 1.00 and 1.04 V·s/cm^2^, respectively, suggest molecular structures similar to Cet and Beh, based on the observed correlation of an increase in migration time and inverse reduced ion mobility. In contrast, the signals 5 and 6 at 1.07 and 1.11 V·s/cm^2^ in Figure 4 do not show such a correlation: While their inverse reduced ion mobilities in the gas phase as separated in the IMS are very close to the signal of Beh (4) (Figure 5C), their migration order in the liquid phase as separated by CE (Figure 5A) is distinctly different. This suggests distinct structural variations, potentially involving heteroatoms, that influence solvent‐analyte interactions more strongly in the liquid phase than in the gas phase, thereby exerting a stronger effect on migration time than drift time. This finding, which also highlights a certain degree of orthogonality between CE and IMS arising from their different separation media, gained further significance as the absence of a distearoylethyl hydroxyethylmonium (DHM) signal contradicted expectations based on product label claims of substantial DHM content. Technical grade DHM was analysed using CE‐IMS and CE‐MS to investigate this discrepancy. The previously unidentified signals were conclusively assigned to monostearoylethyl dihydroxyethylmonium (MHM) and monopalmitoylethyl dihydroxyethylmonium (MHM‐C2) based on CCS value and exact mass analysis, as per the method described in section 3.3. This finding suggests rapid hydrolysis of one or both ester bonds of DHM upon exposure to aqueous solvents, aligning with literature [42].

The presence of MHM‐C2 is likely attributed to palmitic acid as a contamination in the stearic acid typically used for DHM synthesis [45]. Similarly, Signals 2 and 3 in Figure 4 and Figure 5B were assigned as homologues of the Beh cation with a shortened alkyl chain: Based on exact mass and CCS values, the assigned molecular formulas C_23_H_50_N and C_21_H_46_N differ by C_2_H_4_ and C_4_H_8_, respectively, thus being referred to as ‘Beh‐C2’ and ‘Beh‐C4’ in this work. This finding was supported by the presence of identical contaminations in technical grade Beh and is also attributed to typical manufacturing practices.

### 3.3 CCS and Confirmation of Analyte Identities

CCS values for the analytes were determined based on their measured drift times and calculation according to Equation 2 based on the single‐field method [46]:

(2) $$t_{0}=\frac{\beta }{z}\cdot {\left[\frac{m_{i}}{m_{g}+m_{i}}\right]}^{1/2}\cdot \mathrm{CCS}+t_{\mathrm{fix}}$$

*t*_0_ herein is the measured drift time, *t*_fix_ a device‐specific time constant, *m*_i_ and *m*_g_ the molecular masses of the analyte and neutral gas ions, *z* the charge state and *β* an experiment‐specific mobility constant used as slope.

By employing ions with known CCS values [47] as calibrants under identical experimental conditions, the CCS values of unknowns can be calculated based on measured drift time and their assumed molecular weight and charge. Due to their structural similarity to the analytes, TAA ions were used as CCS calibrants and measured in between sample runs. The linear fit was carried out in OriginPro and is shown in Figure S3.

Experimentally determined CCS values were compared to those simulated with three machine learning‐based prediction models: CCSBase, AllCCS2 and SigmaCCS [6, 48, 49]. The SMILES strings of the proposed quaternary ammonium molecules served as input for the first two models, while the readily supplied in‐silico database was used for the latter. The outputs of the models for protonated species were then used for comparison, as these typically gave the best results on average. The AllCCS2 model yielded results with the highest level of agreement.

The differences between measured and predicted CCS values were minimal, ranging from −0.63% to +1.25%, with an average deviation of +0.28%, as shown in Table 2, surpassing the overall median residual error of 1.69% reported for the model's typical performance on DT‐IMS instruments [48].

**TABLE 2:** Comparison of CCS values obtained by measurement‐based calculation and values predicted using the AllCCS2 prediction model.

Species	CCS_Measurement_/Å	CCS_Predicted_/Å	Δ/%
Cet	179.4	180.5	−0.63
Beh‐C4	188.7	189.6	−0.45
Beh‐C2	198.5	197.9	+0.33
Beh	208.3	206.8	+0.73
MHM‐C2	207.2	206.2	+0.45
MHM	217.8	215.1	+1.25

To provide additional evidence for confirming the suspected analyte identifications obtained in this way, supplementary CE‐HR‐QTOF‐MS experiments were performed. Accurate mass determination and comparison of migration order across multiple CE runs provided supporting evidence for structural assignments. The deviation between theoretical exact and measured masses was, on average, in a separation run, +1.8 ppm. The corresponding electropherogram and a list of the observed mass‐to‐charge ratios are shown in Figure S5/Table S3.

### 3.4 Limit of Detection and Quantitation

**FIGURE 6:**
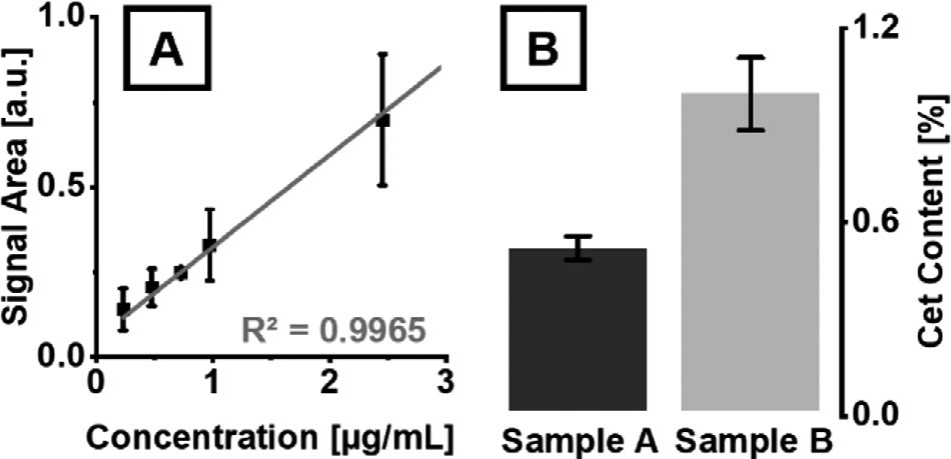
(A) Detail of the calibration curve for Cet (*n* = 3), acquired by CE‐IMS measurements of Cet standard solutions in increasing concentration. (B) Quantitation of Cet content (w/v) in commercial cosmetic products: Sample A: 0.51% ± 0.04% (*n* = 3); Sample B: 0.99% ± 0.11% (*n* = 4).

The limit of detection (LOD) and the calibration curve used for quantitation were determined by performing triplicate CE‐IMS measurements with injections of increasing concentrations of the Cet standard (Figure 6). As a sufficiently pure standard was only available for Cet, this process was not feasible for Beh or DHM. Concentrations reported refer to the concentration of the sample solution. Peak integration was performed using ±13.3 mV·s/cm^2^ and ±5.0 s windows centred on the peak maxima in mobility and migration time domains, respectively. The LOD, calculated from the calibration curve according to standard procedures [50], was established at a concentration of 0.16 µg/mL, corresponding to an injected sample amount of 3 pg Cet. The linear range extended from 0.25 to at least 20 µg/mL with a correlation coefficient (*R* ^2^) of 0.9965. Employing an external calibration, the Cet content in the two hair products was determined. Sample A contained approximately 0.5% (w/v) Cet, while sample B contained about 1.0% (w/v), both complying with current EU regulations [51].

## 4 Conclusion

This study successfully demonstrates the coupling of CE with a high‐resolution IMS using a shifted inlet potential configuration and a nanoflow sheath liquid electrospray interface (ESI). This novel system enabled the rapid analysis of QAC in real‐world samples with minimal sample preparation. The orthogonality between CE and IMS, arising from their different separation media, enhances the analytical performance of the system and provides a complementary approach to conventional techniques. The shifted inlet potential of the IMS facilitates the use of high field strengths for CE separation, resulting in fast analyses. CE‐IMS is a powerful analytical tool and is more affordable than CE‐MS technology. Its cost‐effectiveness, durability and ability to analyse various non‐chromophoric analytes make IMS an excellent detector for CE. Offering structural insights in mixture analysis with minimal instrumental complexity, CE‐IMS provides an exciting, affordable alternative for multidimensional analysis in research and practice.

## Conflicts of Interest

The authors declare no conflicts of interest.

## Supporting information



Supporting Information

## Data Availability

The data underlying the results presented in this study are available upon request from the corresponding author.
