# Supplementary material for: Coupling Capillary Electrophoresis With a Shifted Inlet Potential High‐Resolution Ion Mobility Spectrometer
Source: Electrophoresis. 2025 Apr 28;46(11-12):694–701. doi: 10.1002/elps.8147 (PMC12366286; doi:10.1002/elps.8147)
Supplement: Supplementary file 1 — Supporting Information [file ELPS-46--s001.docx]

Coupling Capillary Electrophoresis with a Shifted Inlet Potential High-Resolution Ion Mobility Spectrometer

K. Welters,^[1]^ C. Thoben,^[2]^ C.-R. Raddatz,^[2]^ F. Schlottmann,^[2]^ S. Zimmermann,^[2]^ D. Belder^[1]*^

[1] Institute of Analytical Chemistry

Leipzig University

Linnéstrasse 3, 04103 Leipzig, Germany

E-mail: belder@uni-leipzig.de

[2] Institute of Electrical Engineering and Measurement Technology

Department of Sensors and Measurement Technology

Leibniz University Hannover

Appelstraße 9a, 30167 Hannover, Germany

Supporting Information

**Table of Contents:**

| Table S1 | Structures of QACs in cosmetic products | p. 2 |
| --- | --- | --- |
| Table S2 | Comparison of theoretical exact and measured m/z-ratios | p. 3 |
| Figure S1 | Photograph of nanoflow sheath liquid ESI in operation | p. 3 |
| Figure S2 | Performance comparison of Triple Tube Sprayer and Nanoflow Sheath Interface | p. 4 |
| Figure S3 | Calibration curve for Cet standard | p. 4 |
| Figure S4 | CCS calibration curve | p. 4 |
| Table S3 | Accuracies of CCS predictions in comparison to measured values | p. 5 |
| Figure S5 | CE-MS electropherogram | p. 5 |

**Table S1**. Structures of QACs in cosmetic products A and B as declared according to INCI ingredient list and of the cationic species as confirmed by analysis.

| Name / PubChem CID | Structure of Declared QACs |
| --- | --- |
| Cetrimonium-chlorid  [CID 8154](https://pubchem.ncbi.nlm.nih.gov/compound/8154) |  |
| Behentrimonium-chlorid  [CID 3014969](https://pubchem.ncbi.nlm.nih.gov/compound/3014969) |  |
| DHM  [CID 21124016](https://pubchem.ncbi.nlm.nih.gov/compound/21124016) |  |
| Name as used /  PubChem CID | Structure of Cationic Species Found, Confirmed by Analysis |
| Cet  [CID 2681](https://pubchem.ncbi.nlm.nih.gov/compound/2681) |  |
| Beh-C4  [CID 8156](https://pubchem.ncbi.nlm.nih.gov/compound/8156) |  |
| Beh-C2  [CID 23768](https://pubchem.ncbi.nlm.nih.gov/compound/23768) |  |
| Beh  [CID 157847](https://pubchem.ncbi.nlm.nih.gov/compound/157847) |  |
| MHM-C2  [CID 169441410](https://pubchem.ncbi.nlm.nih.gov/compound/169441410) |  |
| MHM  [CID 120019](https://pubchem.ncbi.nlm.nih.gov/compound/120019) |  |

**Table S2.** Comparison of theoretical exact and measured masses and their deviation based on CE-MS measurement.

| Cationic Species | Formula | *m/z*_meas_ | *m/z*_theo_ | Δ*m*/*z* / ppm |
| --- | --- | --- | --- | --- |
| Cet | C_19_H_42_N^+^ | 284.3332 | 284.3317 | 5.2 |
| Beh-C4 | C_21_H_46_N^+^ | 312.3621 | 312.3630 | -3.0 |
| Beh-C2 | C_23_H_50_N^+^ | 340.3947 | 340.3943 | 1.1 |
| Beh | C_25_H_54_N^+^ | 368.4275 | 368.4256 | 5.1 |
| MHM-C2 | C_23_H_48_NO_4_^+^ | 402.3576 | 402.3583 | -1.8 |
| MHM | C_25_H_52_NO_4_^+^ | 430.3874 | 430.3896 | -5.2 |


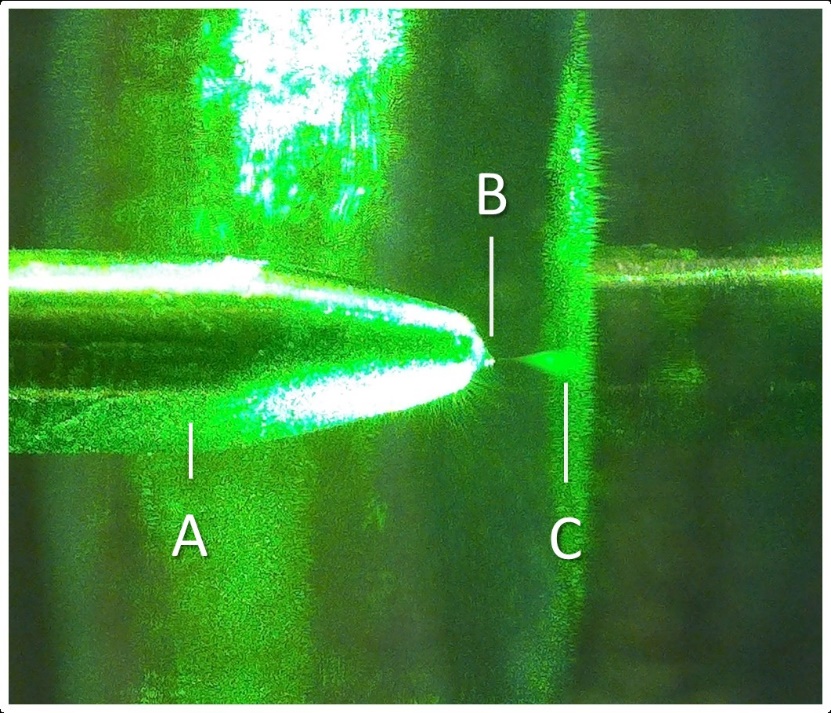


**Figure S1**. CE-ESI interface with green lighting for spray visualization during operation of the nanoflow sheath liquid electrospray. **A**: Outer stainless-steel sheath capillary, **B**: Tapered fused silica capillary, **C**: ESI spray cone entering the IMS inlet.

|  |  |
| --- | --- |
| **Figure S2.** Comparison of signal intensities and RSDs depending on sheath liquid flow rate between the triple tube sprayer in the nanoflow mode (without sheath gas) and the homebuilt nanoflow sheath liquid interface used in this work. The interfaces were positioned centered and at a distance of 3 mm between the IMS inlet and the capillary tip. The protrusion of the tapered fused silica capillary was set at about 100 µm for both interfaces. The signals were obtained by continuous electrokinetic injection of 10 µM tetrahexylammonium bromide and 20-sec intervals of continuous sheath flow rates (= ~200 spectra per flow rate datapoint). CE parameter: Separation voltage: +30.0 kV, capillary length: 68 cm, sheath flow: 8/2 ACN/H_2_O (v/v) at variable flow rates, BGE: 8/2 ACN/H_2_O (v/v) + 10 µM tetrahexylammonium bromide, ESI voltage: +3.5 kV. | |

|  |  |
| --- | --- |
| **Figure S3.** Calibration curve for Cet standards measured using triplicate CE-IMS injections of increasing concentrations. | **Figure S4.** Cross section calibration curve based on drift times of tetrabutyl-, tetrahexyl- and tetraoctylammonium cations. |

**Table S3.** CCS values as calculated from measurement and calibration based on tetraalkylammonium salts (CCS_Meas_) or simulated by three prediction models for the protonated species of the cations (CCS_Sim_). CCS values for SigmaCCS were taken from the in-silico database. The deviation Δ is calculated as ((CCS_Meas_/CCS_Sim_)-1)*100 from the unrounded values. As the rather exotic cationic species MHM-C2 was not included in the otherwise very extensive SigmaCCS in-silico database, this value was omitted.

| Cationic Species | CCS_Meas_ / Å | CCS_Sim_ / Å | | | | | |
| --- | --- | --- | --- | --- | --- | --- | --- |
|  |  | AllCCS2 | Δ / % | CCSBase | Δ / % | SigmaCCS | Δ / % |
| Cet | 179.4 | 180.5 | -0.63 | 183.8 | -2.41 | 190.0 | -5.60 |
| Beh-C4 | 188.7 | 189.6 | -0.45 | 193.4 | -2.40 | 199.0 | -5.15 |
| Beh-C2 | 198.5 | 197.9 | +0.33 | 202.9 | -2.16 | 208.4 | -4.74 |
| Beh | 208.3 | 206.8 | +0.73 | 212.2 | -1.82 | 215.9 | -3.51 |
| MHM-C2 | 207.2 | 206.2 | +0.45 | 214.6 | -3.47 | x | x |
| MHM | 217.8 | 215.1 | +1.25 | 224.0 | -2.76 | 218.3 | -0.22 |

**Figure S5.** Electropherogram of CE-MS measurement with EIC mass traces. Traces offset by 5E4 counts.
